# Dyadic Work in Conjunction With Electroconvulsive Therapy in a 12-Year-Old Female With Autism Spectrum Disorder and Catatonia

**DOI:** 10.31486/toj.23.0095

**Published:** 2024

**Authors:** Ryan Meeder, Autumn Peterson, Hannah Reynard, Laura Andersen

**Affiliations:** Department of Child and Adolescent Psychiatry, University of Michigan, Ann Arbor, MI

**Keywords:** *Autism spectrum disorder*, *behavior therapy*, *catatonia*, *electroconvulsive therapy*

## Abstract

**Background:** Attachment-based interventions have been extensively studied in neurotypical patient populations. In neurodivergent patient populations, however, emphasis on and current research into attachment-based interventions are centered on early childhood. Minimal research has been conducted in school-aged children with autism spectrum disorder (ASD), and even less research has focused on attachment-based interventions for children with significant comorbidities such as catatonia.

**Case Report:** We present the case of a 12-year-old female that involved dyadic work in conjunction with biologic interventions for the treatment of ASD and catatonia. Psychosocial interventions were centered on an attachment-based framework and behavioral skills training that incorporated elements of parent management training. We observed and tracked the patient's uncooperativeness, underproductive speech, emotional withdrawal, and anxiety via the Brief Psychiatric Rating Scale for Children. Attachment- and behavioral-based interventions in conjunction with psychotropic medications and electroconvulsive therapy resulted in improvements.

**Conclusion:** This case illustrates the potential advantages that attachment- and behavioral-based psychotherapeutic interventions can confer in complex cases involving neurodivergent patients. The case also highlights the lack of current research into and understanding of attachment theory in children and adolescents with ASD. Research is needed into the role of attachment-based interventions in patients with ASD and other psychiatric comorbidities, particularly in patient populations beyond preschool age. Initiating nonbiologic interventions in conjunction with biologic interventions may also enhance outcomes and warrants further investigation.

## INTRODUCTION

Attachment is a neurobiologic process consisting of the dyadic relationship and bond between a child and their primary caregivers that underpins social and emotional development throughout the lifespan. Given that attachment disorders and attachment-related difficulties have long been studied in neurotypical populations, a 2021 publication suggested that addressing attachment in neurodivergent patient populations, specifically in patients with autism spectrum disorder (ASD), may improve outcomes as attachment-based interventions have been shown to do in neurotypical populations.^1^ Findings of a study examining the sensitivity of mothers with children with ASD indicated that more sensitive parents increase the likelihood of secure attachment with their children.^2^ The interplay between the biologically based impairments seen in ASD and environmental factors, such as caregiver-child interactions and low levels of maternal psychosocial stress, also helps mitigate attachment formation.^3^ Furthermore, secure attachment has long been associated with healthy development of social relationships, emotional and behavioral regulation, and curiosity, while insecure attachment styles have been associated with the development of psychopathology.^4^ A comparative study by Teague et al published in 2018 suggested that secure attachment styles in children with ASD improve outcomes relative to children with insecure attachment styles in areas such as joint attention, complex play, emotional regulation, and education.^5^

Compared to neurotypical populations, children with ASD are at increased risk of catatonia, which can further complicate treatment.^6,7^ Patients with ASD have a high presence of catatonic features, with a report from 2006 indicating that 17% of adolescents and adults across 3 studies demonstrated severe catatonia,^8^ and the *Diagnostic and Statistical Manual of Mental Disorders, Fifth Edition* recognizes catatonia as a possible feature of ASD. Treatment of catatonia is primarily biologic, via electroconvulsive therapy (ECT) and psychotropic medications.^9^ A 2021 meta-analysis found that antipsychotics were used in 27% to 100% of individuals with ASD who developed catatonic features, benzodiazepines were used in 55.6% to 95.5%, and ECT was used in 22 of 1,534 individuals; however, because of insufficient data, the relationship between therapeutic, cognitive, and psychopathologic interventions related to ASD and catatonia could not be evaluated.^10^ Psychotherapeutic interventions for the treatment of catatonia are not often initiated until the patient's symptoms of catatonia are resolved or the patient has the capacity to engage in treatment.^11^

We present a case of a 12-year-old female with ASD and catatonia with insecure attachment whose treatment outcome was improved with the addition of behavioral- and dyadic-based interventions on an inpatient unit while receiving an index course of bilateral ECT. This case illustrates the importance of increasing awareness about initiating attachment- and behavioral-based work in children and adolescents with ASD, as it can enhance treatment outcomes and should not be deferred until biologic interventions are completed.

## CASE REPORT

A 12-year-old Asian female inpatient with a medical history of Graves disease status post thyroidectomy, hypoparathyroidism, ASD, and catatonia had the following notable presenting symptoms: insomnia, mood lability, underproductive speech, anxiety, emotional withdrawal, uncooperativeness, intermittent mutism, intermittent incontinence, altered mental status, stereotypies, negativism, and odd mannerisms. Her presentation was complex, given the juxtaposition of biologic and functional symptoms that often overlapped and were exacerbated by parental enmeshment in the sick role and inadvertent reinforcement of maladaptive behaviors as a result of parental distress. Prolonged observation of the patient's behavior, communication, and relational patterns showed that her regression (speech, engagement, and independent initiation of tasks and daily living skills) and often her symptoms characteristic of ASD and catatonia were most pronounced in her parents’ presence, particularly during reunifications and separations. Additionally, the patient's affect appeared heightened, and she exhibited intrusive boundaries and limited capacity for autonomous behavior when interacting with her parents. The patient's regression in functionality and social communication was reinforced by parental attention and avoidance of limit setting. The social-communication impairments often characteristic of ASD were interfering with the dyad's capacity to anticipate and interpret each other's emotions, motives, and behaviors, thereby limiting reciprocal, attuned, and rewarding interactions during the patient's development.

Additionally, the parents’ stress (severely ill child, lack of social support, marital conflict, financial constraints, and several barriers to accessing outpatient treatment), emotional reactivity, and over-enmeshed parenting approach compromised their capacity to be consistently attuned and responsive and to set developmentally appropriate limits. Parental grief and sadness regarding the child's illness also inhibited security within the dyad.

The patient's psychiatric history was notable for 1 inpatient psychiatric admission within the prior year for altered mental status. Family history was notable for younger twin brothers with ASD, and the family reported probable ASD on the paternal side. Prior failed medication trials included olanzapine, risperidone, clonidine, guanfacine, methylphenidate, zolpidem, and hydroxyzine. The patient had failed trials of high-dose lorazepam (up to 17 mg daily) due to disinhibition and poor treatment response, prompting the treatment team to pursue ECT. Following the taper of high-dose lorazepam, the patient was started on a course of bilateral ECT 3 times weekly for treatment-resistant, agitated catatonia. While she was receiving ECT, the patient was administered lorazepam 1 mg 3 times daily and lithium 900 mg daily.

The index course of bilateral ECT targeting agitated catatonia was in conjunction with a multidisciplinary team treatment approach involving child and adolescent psychiatrists; board-certified behavior analyst; clinical social worker; learning specialist; activity therapists; and nurses targeting symptoms related to catatonia, ASD, and parent-child attachment. To maintain consistency in evaluating treatment response over time, a clinician who was certified in attachment-based family therapy and parent management training and was an Infant Mental Health Specialist with level I endorsement led the dyadic sessions throughout treatment.

We implemented an attachment-oriented approach in conjunction with behavioral skills training to promote positive parent-child interactions and deescalate distress within the dyad. Dyadic and behavioral work was simultaneously facilitated between the patient and her primary caregiver, her mother, in 9 dyadic sessions and 4 parent-only sessions. Relational interventions emphasized parent-child attunement, caregiver synchrony, sensitivity, emotional availability, and responsiveness. Behavioral-based interventions included parent skills training focused on appropriate parent-child interactions and behavior management strategies. Parent training was carried out in experiential and structured sessions in a behavioral skills training format (instruction, modeling, rehearsal, and feedback) and incorporated elements of parent management training.

Attachment-based interventions in tandem with behavioral-based interventions facilitated the caregiver's capacity for emotional attunement and support of autonomy development, reflective functioning, and sensitivity and allowed the child to learn to verbally express her internal experience, learn to trust her parents’ response to her complex needs, regulate difficult emotions, tolerate conflict, and negotiate autonomy. We emphasized identifying the function of disruptive behaviors and maladaptive patterns of relational interactions, addressing problem-maintaining patterns within the family system, deescalating parent-child reactivity, enforcing boundaries and developmentally appropriate limits, implementing routine and structure, building caregiver emotional regulation, and promoting independent engagement in daily living skills and tasks.

These dyadic- and behavioral-based interventions, in conjunction with ECT, produced a robust response in terms of overall functionality, independence, and secure parent-child interactions. Clinician and behavioral analyst impressions indicated a progressive increase in mobility, reduction in delayed responses, increase in engagement in activities, and global improvement in independence. Family sessions and parent coaching appeared to reduce reinforcement of functional loss and maintenance of the sick role. We observed a high level of parental emotional distress and subsequent intrusiveness during structured parent-child play interactions prior to the initiation of attachment-based interventions. Over the course of the family sessions, we observed a substantial reduction in overprotective parenting behaviors and caregiver distress compared to data collected during parent-child interactions upon admission. The child's primary caregiver appeared more attuned and responsive, and the patient exhibited increased autonomy and developmentally appropriate communication and behavior with her caregiver. As the relationship improved, the family demonstrated increased capacity to engage in planning for the transition to outpatient biologic, behavioral, and relational interventions.

**Figure 1.**
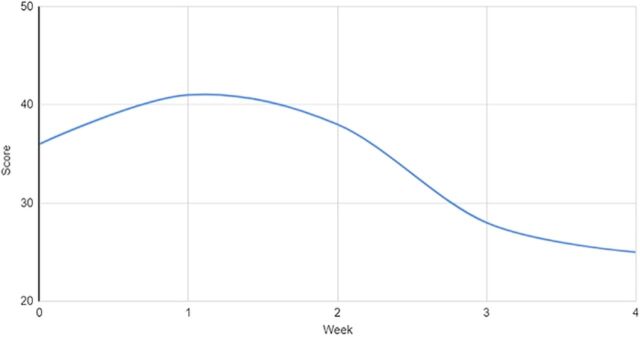
Brief Psychiatric Rating Scale for Children weekly total scores during weeks 0 to 4 of the patient's treatment. Scores are generated from 18 discrete subcategories scored on a scale of 0 to 7: not assessed (0), not present (1), very mild (2), mild (3), moderate (4), moderately severe (5), severe (6), and extremely severe (7). The patient's peak score of 41 in week 1 decreased over time to 25 by week 4, a 39% reduction.

**Figure 2.**
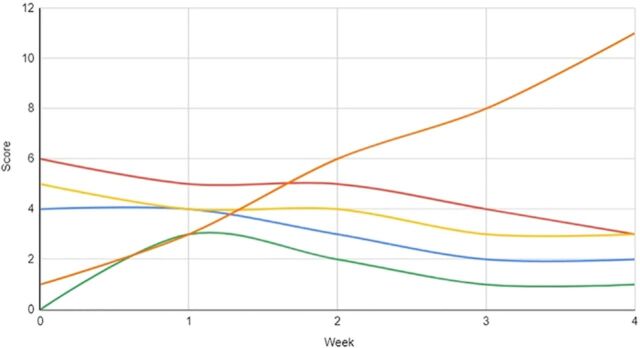
Brief Psychiatric Rating Scale for Children subcategory weekly scores during weeks 0 to 4 of the patient's treatment: uncooperativeness (blue line), underproductive speech (red line), emotional withdrawal (yellow line), and anxiety (green line). Subcategory scores range from 0 to 7. The number of dyadic sessions over time is graphed in orange. As the number of dyadic sessions increased throughout admission in conjunction with biologic interventions, downward trends were observed in the Brief Psychiatric Rating Scale for Children subcategories.

**Figure 3.**
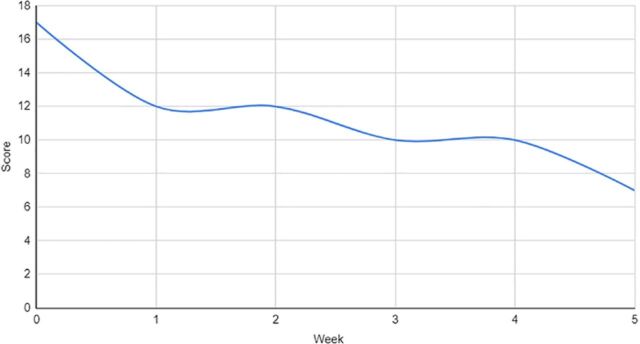
Bush-Francis Catatonia Rating Scale weekly scores during weeks 0 to 5 of the patient's treatment. Bush-Francis Catatonia Rating Scale subcategory scores range from 0 to 3, with higher scores indicating worsening severity of symptoms. The patient's peak score of 17 at the start of treatment decreased over time to 7 by week 5.

The patient underwent 15 courses of ECT while an inpatient. ECT and psychotropic medications were maintained consistently throughout this interval of approximately 35 days. Her response to interventions was measured via the Bush-Francis Catatonia Rating Scale and the Brief Psychiatric Rating Scale for Children throughout the duration of dyadic work in conjunction with biologic interventions. Her response to treatment was trended over time and is graphed in Figures 1, 2, and 3. The patient was discharged home with her parents and was followed longitudinally in the outpatient setting for medication management, monitoring of response and frequency of continued ECT, and initiation of applied behavior analysis therapy. The family was also provided with resources to pursue outpatient dyadic-based work centered around attachment.

## DISCUSSION

The role of attachment in family units with neurodivergent children is complex, particularly when comorbidities are also present, such as in cases of ASD with catatonia.^12^ In 2014, DeJong et al published a systematic review of 22 articles discussing the treatment of 28 patients with ASD and features of catatonia.^13^ Only 3 papers mentioned the use of behavioral interventions. The studies demonstrated positive outcomes in symptom reduction with behavioral treatments that included providing psychoeducation for carers, reducing stress, encouraging engagement in activities, using prompting, and maintaining routine; however, the results were inconclusive given the limited objective data and the presence of several confounding variables (eg, simultaneous biologic interventions, therapeutic benefit of the inpatient milieu).^13^ A case report from 2021 highlighted the benefit of behavioral interventions in a neurodevelopmentally delayed adult patient receiving ECT for catatonia during a prolonged inpatient admission.^14^ We searched for papers reporting attachment-based interventions in patients with comorbid ASD and catatonia and papers examining the role of attachment-promoting interventions for pediatric inpatients with neurodevelopmental delay but identified no publications.

The few articles regarding autism and attachment-based interventions all have a primary focus on early childhood age groups.^1^ A randomized controlled trial using the Video-feedback Intervention to promote Positive Parenting adapted to Autism (VIPP-AUTI) for 5 sessions found attachment-based interventions to be partly effective on parental interactive behavior, reported feelings of child rearing, and child developmental skills.^15^ Notable outcomes were a decrease in observed parental intrusiveness towards the child (caregiver behavior undermining the child's autonomy) and an increase in parental self-efficacy. In 2014, Siller et al investigated intervention effects on attachment-related outcomes in children with ASD through evaluating the efficacy of the Focused Playtime Intervention.^16^ Results demonstrated significant increases in attachment-related behaviors compared to the control group. In 2021, Kubo et al completed a randomized controlled trial measuring the effectiveness of Circle of Security–Parenting, an attachment-based parenting program, that showed improved parental self-efficacy and mental health (with the largest improvement in the categories of empathy and understanding) and decreased parental reports of emotional and behavioral problems in their children with ASD.^17^ Of all the aforementioned studies, the sample sizes were small and treatment measures were often subjective. Additionally, the studies were time-limited and patient populations were not followed longitudinally.

In neurodevelopmentally delayed children compared to neurotypical children, parental emotional overinvolvement is associated with a higher presence of disruptive behaviors and expression of ASD features. Corrective attachment experiences and increased positive parent-child interactions often improve the parent-child relationship and decrease disruptive behaviors and symptoms characteristic of ASD.^18^

Parents of children with ASD report higher levels of parenting-related stress and psychological stress than parents of typically developing children.^19^ We facilitated 4 parent-only sessions to provide the mother of our patient with individual support given the high level of parental stress, caregiver fatigue, and grief and loss related to the child's regression in functioning. Parent training literature has established that a high level of parental stress is a significant barrier to learning skills and adhering to the implementation of behavioral-based interventions.^17^ Parental stress often contributes to inadvertently reinforcing and exacerbating disruptive behavior via higher levels of caregiver attention.^18^ As demonstrated by Circle of Security–Parenting, parents who participated in a parent support component (protected time for the parent to express concerns and elicit support related to parental stress) to augment their participation in behavioral-based intervention work demonstrated reduced stress levels and greater child behavior change compared to parents who did not participate in parent-only sessions.^17^

Parental overaccommodation and enmeshment can exacerbate and reinforce functional symptoms that often worsen health outcomes and hinder progress.^20^ Parental attention and avoidance of limit setting due to parental distress may be secondary gains from inadvertent reinforcement of functional behavior.^20^ Behavioral-based interventions can help to eliminate secondary gains from the sick role and promote improved functioning and health-focused behaviors.^21^ Family engagement in the treatment process can also enhance symptom reduction, sustained progress in treatment, and global improvement in functioning.^22^

Cross-cultural studies have shown some variation in attachment behavior and caregiving practices.^23^ The family in this case was Asian, and much of the attachment theory research and interventions originated from studying white, middle-class populations.^23^ However, attachment theory and the correlation between parental sensitivity and attachment security have been supported by studies of individuals of mixed ethnic and socioeconomic demographics, demonstrating that attachment is a universal biologic process.^23^ One study of families with children with ASD reported family members feeling grief as intense as that experienced upon the death of a family member when receiving a diagnosis of ASD and that the diagnosis signified the loss of a previously healthy child.^24^ Additionally, compared to neurotypical individuals, individuals with ASD are often more socially, financially, and emotionally dependent on their caregivers and family systems across the developmental lifespan which can result in a high burden for caregivers.^25,26^

While attachment- and behavioral-based interventions appeared to improve treatment response in our patient, our case has several limitations and possible confounding factors. As demonstrated in the figures, we observed improvements in symptoms that overlap between ASD and catatonia. Assessing the response to psychosocial interventions administered in conjunction with biologic interventions is difficult because of symptom overlap and the complex changes that biologic interventions such as ECT can generate. More tailored and validated clinical tools measuring attachment would limit subjective findings. Additionally, while 1 clinician completed the rating scales included in this report and this individual has no affiliation with its publication, the rating scales are subjective in nature. Observed improvements could also be considered multifactorial, given that several simultaneous interventions were provided (psychotropic medications, ECT, dyadic work, inpatient treatment); however, research on inpatient interventions outside of biologic treatments is limited.

## CONCLUSION

This case illustrates the potential advantages that attachment- and behavioral-based psychotherapeutic interventions can confer in complex cases involving neurodivergent patients. The case also highlights the lack of current research into and understanding of attachment theory in children and adolescents with ASD. Not only is the existing literature scarce, but it also lacks strong evidence demonstrating efficacy and effectiveness of attachment-based interventions in children with ASD, particularly in school-aged children. Of the case studies reviewed, treatment procedures were often opaque, and outcomes were based on subjective data, indicating the need for controlled treatment trials. Further research is needed regarding the role of attachment-based interventions in patients with ASD and other psychiatric comorbidities, particularly in patient populations beyond preschool age. Initiating nonbiologic interventions in conjunction with biologic interventions may also enhance outcomes and warrants further investigation.

## Notes

*This article meets the Accreditation Council for Graduate Medical Education and the American Board of Medical Specialties Maintenance of Certification competencies for Patient Care and Medical Knowledge.*
